# The ecological mechanism of digital technology integration: a comparative study of psychological and institutional support among university teachers in China and Kazakhstan

**DOI:** 10.3389/fpsyg.2026.1959868

**Published:** 2026-09-22

**Authors:** Chunling Wang, Xiaogang Jin, Duisenbayev Abay, Zhan Zhou

**Affiliations:** 1 Faculty of Philosophy and Political Science, Al- Farabi Kazakh National University, Almaty, Kazakhstan; 2 Continuing Education College, Chang'an University, Xi'an, China; 3 Department of Pedagogy and Educational Management, Al-Farabi Kazakh National University, Almaty, Kazakhstan

**Keywords:** cross-cultural comparison, digital self-efficacy, digital technology integration, higher education ecosystem, institutional support, technology anxiety

## Abstract

**Objective:**

To explore the covariant ecological network of psychological cognition and macro-institutional environment in the process of digital technology integration among university teachers, and to compare the cross-cultural characteristics and differences between China and Kazakhstan.

**Methods:**

Using a convenience sampling method, a cross-sectional survey design was adopted to collect questionnaire data from 1,650 in-service teachers from public universities in China and Kazakhstan (907 from China, 743 from Kazakhstan). Structural Equation Modeling (SEM) was applied to measure the direct and indirect associations among variables, and Multi-Group Analysis (MG-SEM) was used to test the cross-cultural heterogeneity of this structural network.

**Results:**

The data confirmed: (1) Digital self-efficacy is significantly and positively correlated with the frequency of technology integration behavior; (2) Technology anxiety acts as a significant mediator, wherein higher digital self-efficacy alleviates anxiety and subsequently increases technology integration frequency; (3) Although institutional support does not significantly moderate the internal psychological cognitive processes, it acts as an independent system-level resource that directly and significantly promotes higher levels of technology integration; (4) The ecological network exhibits cross-cultural invariance (path heterogeneity between groups is not significant), but teachers in Kazakhstan report a significantly higher perceived mean of institutional support than their Chinese counterparts.

**Conclusion:**

The digital transformation of higher education cannot solely rely on the accumulation of hardware, but should move towards a sustainable ecological model of “psychological adaptation-institutional inclusion” two-way synergy. This empirical model provides data coordinates for the formulation of intelligent supporting policies in the “Belt and Road” multilateral education network.

## 1 Introduction

The digital transformation of higher education has gradually evolved from the popularization of equipment to an ecological stage characterized by human-machine collaboration and intelligent algorithms (such as generative artificial intelligence and adaptive learning systems) (Celik, 2023; Eltaiba et al., 2025; Timotheou et al., 2023). This profound transformation extends the boundaries of traditional teaching practices by driving a fundamental pedagogical transformation rather than merely supplementing hardware. As recently highlighted by Liang et al. (2024) and Farrokhnia et al. (2024), this shift requires a fundamental realignment of pedagogical strategies. In this transformation network, university teachers act as executors translating technological tools into enhanced teaching efficacy (i.e., the successful realization of pedagogical goals such as interactive lesson design and personalized student assessment), and their psychological adaptation to the new environment is highly correlated with the ultimate depth and frequency of their technological application in teaching practices (Ertmer and Ottenbreit-Leftwich, 2010). Past empirical data indicates that the effective retention of technology in the educational field depends not only on the perfection of software tools but also on teachers’ individual cognition and the structural characteristics of the organizations they belong to (Altmiller and Pepe, 2022; Mekheimer, 2025; Teo, 2011).

To better understand this phenomenon, it is essential to move beyond isolated technology acceptance models. Instead, researchers should conceptualize teachers’ technology integration as a complex ecological process embedded within multi-layered organizational and psychological systems (Blannin et al., 2025; Zhao and Watterston, 2021). Delving into the essence of this research topic, Socio-Technical Systems (STS) theory posits that technological adoption is not merely an operational shift but a continuous negotiation between macro-institutional structures and micro-psychological states (Bearman et al., 2023; Dwivedi et al., 2023). At the macro level, universities, as entities for policy formulation and resource allocation, create an external environment for teachers’ digitalization through their advocated institutional frameworks and fault-tolerant cultures (Institutional Support, IS) (Alshammari and Alenezi, 2023; Bao, 2020). Ascending to the micro level, the Digital Self-Efficacy (DSE) and accompanying Technology Anxiety (TA) exhibited by individuals when facing technological changes constitute the internal cognitive antecedents for information processing and behavioral output (Ertmer et al., 2012).

Despite these fragmented theoretical advances, defining research gaps remain highly visible. First, existing theoretical reviews often treat institutional climate and psychological traits separately (Inan and Lowther, 2010), failing to thoroughly explore their multidimensional covariance within a unified, interactive ecosystem model. Second, the socio-technical buffering role of institutions in mitigating deep-seated negative emotions, specifically under advanced AI-driven contexts, remains an underexplored essential mechanism.

In examining the universality and heterogeneity of this ecological mechanism, macro-institutional differences at the cross-national level provide suitable theoretical observation contexts (Cazan et al., 2024). Taking China and Kazakhstan as examples, their selection is theoretically justified by their highly distinct institutional cultures and digital evolution trajectories. Although both countries are located within the higher education cooperation network under the “Belt and Road” initiative, they exhibit distinct organizational characteristics in the policy paths and evolutionary pace of advancing education informatization. Higher education in China has experienced high-intensity, top-down penetration (Zhou et al., 2020), with a high popularization rate of various intelligent educational administration and online learning platforms. In contrast, Kazakhstan is at a critical juncture in accelerating the implementation of digital transformation strategies (such as “Digital Kazakhstan”), and its grassroots management and fault-tolerance systems are still in the exploratory stage (Dzhanegizova, 2024). Incorporating these two countries—which share geopolitical cooperation attributes but have differentials in informatization stages—into the same observation model not only helps to test the cross-cultural consistency of the psychological chain (from self-efficacy to technology anxiety) (Kirkman et al., 2006) but also validates the moderating characteristics of institutional support within a broader policy spectrum. This comparative approach fundamentally tests whether ecological coping mechanisms are universally human or culturally contingent.

Based on the above background, this study designed a cross-sectional empirical survey targeting university teachers in China and Kazakhstan, aiming to systematically explore the ecological mechanism of digital technology integration. Specifically, this study focuses on evaluating: (1) the statistical association between digital self-efficacy and technology integration behavior; (2) the mediating role of technology anxiety therein; (3) the main effect association of perceived institutional support and its moderating function when linking psychological beliefs; (4) the model path differences between China and Kazakhstan based on multi-group analysis. This study not only provides quantitative supplements for educational technology ecosystem theory but also offers data references for university administrators formulating supporting policies for intelligent transformation.

## 2 Theoretical basis and research hypotheses

In exploring the paths of digital teaching behaviors, Socio-Technical Systems Theory and Ecosystem Approaches provide a systematic explanatory framework. Under this framework, individual behavior is the mapping of complex interactions between psychological cognition and the institutional environment. This study conceptualizes digital self-efficacy and technology anxiety as a “psychological cognition-emotional response” chain, and defines institutional support as a peripheral environmental moderating factor. Based on the covariance logic of cross-sectional data, this study aims to reveal how the aforementioned multidimensional traits jointly associate with the frequency of technology integration behavior.

### 2.1 The theoretical linkage between digital self-efficacy and technology integration behavior

Digital self-efficacy stems from Bandura’s Social Cognitive Theory (Bandura, 1977). This study defines it as university teachers’ capability assessment of relying on their own abilities to master and apply advanced digital tools to achieve teaching goals (Hatlevik, 2017). Existing research indicates that self-efficacy is the core antecedent for individuals to maintain goal-directed behaviors when facing uncertainty (Abbitt, 2011).

In a digital education environment with extremely high update frequencies, high-frequency technology integration means teachers must frequently undertake the professional risks of reconstructing traditional classrooms (Khlaif et al., 2025). According to social cognitive logic, individuals with high digital self-efficacy are more likely to view technological iterations as innovative opportunities rather than pure occupational threats. They demonstrate stronger psychological tolerance when facing the learning curves of software systems (Sun and Yoon, 2025), thereby integrating advanced teaching functions more frequently and multidimensionally. Based on the assumption of consistency between beliefs and explicit behaviors, the following hypothesis is proposed:

*H1*: Digital self-efficacy is significantly and positively correlated with technology integration behavior.

### 2.2 The indirect associative role of technology anxiety

The Technostress framework suggests that technology-related behavioral manifestations highly covary with negative emotional responses (Ragu-Nathan et al., 2008). Technology anxiety refers to the sense of resistance and exhaustion individuals experience when operating or anticipating the use of complex technologies, serving as a typical negative manifestation in digital survival (Edú-Valsania et al., 2022; Rasool et al., 2022).

In a multi-variable transmission network, technology anxiety is hypothesized as a mediating variable connecting self-concept and practical output. A lack of confidence often accompanies panic about environmental uncontrollability; conversely, a high level of digital self-efficacy can stabilize evaluations and mitigate negative emotional arousal, with the two showing a structural negative correlation (Mushtaque et al., 2022; Zivi et al., 2025). Simultaneously, teachers trapped in a high-anxiety state fall into a state of cognitive resource depletion (Yang et al., 2025), reducing the cognitive capacity available to explore integration solutions and thereby lowering practice frequency (Zhang, 2023). In short, high self-efficacy typically corresponds to a lower anxiety baseline, which in turn co-occurs with higher-frequency technological practices. Accordingly, the following hypothesis is proposed:

*H2*: Technology anxiety plays a significant indirect associative role between digital self-efficacy and technology integration behavior, such that higher digital self-efficacy is associated with lower technology anxiety, which in turn corresponds to more frequent integration behavior.

### 2.3 Environmental synergy and conditional buffering characteristics of institutional support

Based on Organizational Support Theory, employees’ perception of the organization’s inclusion of their wellbeing is closely related to their behavioral patterns (Venkatesh et al., 2003). In the context of educational technology adoption, institutional support encompassing hardware connectivity, technical assistance, and fault-tolerant mechanisms is not only the foundational environment where integration occurs but also participates as an external condition in regulating the relationship network between micro-psychology and practice (Nascimento et al., 2025).

As an independent environmental resource, the organizational support framework is directly associated with the breadth of technology integration. When teachers perceive more resource inclination and policy inclusion, it is often accompanied by more frequent technological exploration (Kundu et al., 2021; Saleem et al., 2024). Consequently, specific environmental performance levels and technology integration behaviors exhibit a common upward-trending characteristic.

At the conditional buffering level, institutional support is assigned a two-way moderating logic. For high technology anxiety groups, the lack of peripheral support networks easily induces technology avoidance tendencies (Khlaif et al., 2023); whereas a well-established institutional support system is expected to weaken the connection strength between anxiety and avoidance by providing a pool of available resources. At the belief conversion level, teachers in high-support environments have a smoother pathway extending self-efficacy to behavior, meaning institutional mechanisms can strengthen the positive coexistence of self-confidence and practice. Accordingly, we propose:

*H3a*: The level of institutional support is significantly and positively correlated with technology integration behavior.

*H3b*: Institutional support exerts a negative buffering effect; under higher institutional support, the negative association between technology anxiety and technology integration behavior is significantly weakened.

*H3c*: Institutional support exerts a positive reinforcement effect; in samples with high support, the positive association between digital self-efficacy and technology integration behavior is tighter.

### 2.4 Cross-national comparative perspective

Technology and its derivative mechanisms possess not only organizational heterogeneity but are also reflections of macro-educational policies. The higher education systems in China and Kazakhstan exhibit systematic differences in internal resource allocation, evaluation weights, and digitalization accumulation. For example, digitalization in Chinese universities is often accompanied by strong policy directives and high-intensity hardware popularization; while Kazakhstan is mostly exploring standardized paths for blended teaching.

Due to objective differences in management systems, cross-national comparisons provide natural contexts to test the boundary conditions of the model. Theoretically, macro-environmental differences may map onto structural heterogeneity in the linkages between psychology and behavior. Under a performance-oriented model, institutional support may be alienated into invisible “assessment-driven pressure” (Siddiqi et al., 2025), leading to a depreciation in its expected utility of alleviating anxiety. In the early stages of transformational exploration, subtle fault-tolerant support is more likely to transmit sensitive and positive environmental signals to individuals.

However, from a competitive perspective, human foundational cognitive mechanisms for coping with technological uncertainty (such as the transmission from self-efficacy to stress/anxiety) often transcend policy restrictions, demonstrating structural isomorphism. To test whether the interactive network nested with institutional and psychological elements exhibits policy specificity or cross-cultural invariance, this study proposes an exploratory comparative hypothesis:

*H4*: In the complete structural equation model, there is significant inter-group heterogeneity in the core path coefficients between the teacher samples of China and Kazakhstan.

Synthesizing the deductive logic of the above Socio-Technical Systems Theory and Ecosystem Approaches, the theoretical hypothesis model of the ecological mechanism of digital technology integration in higher education constructed in this study is shown in Figure 1.

**Figure 1:**
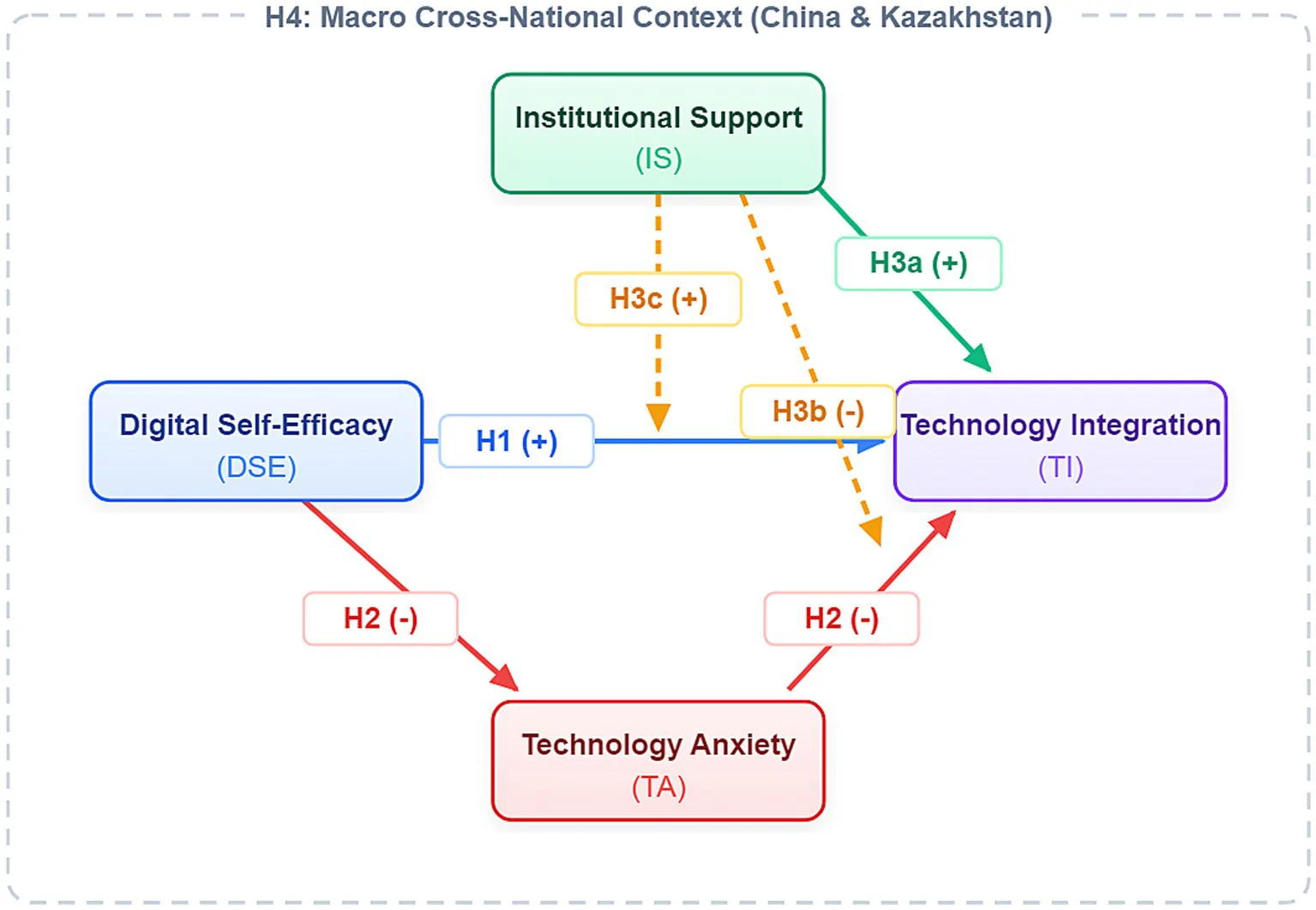
Theoretical hypothesis model of the ecological mechanism of digital technology integration.

## 3 Research methods

### 3.1 Participants and procedure design

This study adopted a cross-sectional survey design, distributing questionnaires to in-service public university teachers in China and Kazakhstan via online platforms. Given the heterogeneity of cross-national samples in the language comprehension dimension, the questionnaire compilation strictly followed a “translation-back translation” procedure. Parallel initial translations were conducted by two experts with educational backgrounds and Chinese-Russian bilingual capabilities, followed by back-translation executed by an independent language expert not involved in the initial translation. By comparing the semantics of the original and back-translated versions, the research team fine-tuned some expressions to control cross-cultural measurement errors.

Data collection was concentrated from March to June 2025, utilizing a convenience sampling method to adequately capture the demographic and institutional diversity across both countries. To ensure the reliability of this large-scale sample, the survey deployment was systematically coordinated through the administrative systems of partnering universities. By collaborating directly with various teaching departments, the digital questionnaires were accurately routed to eligible full-time faculty members actively engaged in teaching during the current academic year. Participants were required to read an informed consent form and confirm electronically before answering. To control for Common Method Bias (CMB), in addition to following anonymity and confidentiality principles, the procedural design deliberately separated measurement formats: the dependent variable (technology integration behavior) used a frequency scale, consciously distinguishing it from other agreement-based psychological and environmental variables (Podsakoff et al., 2003).

In the data preprocessing stage, reverse attention check items had a small proportion (about 7.8%) of incorrect responses. Given that traits investigated in this study, such as “technology anxiety,” belong to high cognitive load variables, to maximally retain the natural variance (Ecological Variance) of teachers in real educational ecosystems, this study avoided data homogenization caused by over-trimming. After excluding patterned responses and severe missing values from the 1,750 initially distributed surveys, the valid questionnaires ultimately included in the core analysis set totaled 1,650 (an effective recovery rate of 94.3%), of which the Chinese sample accounted for 55.0% (*n* = 907) and the Kazakhstan sample accounted for 45.0% (*n* = 743). Subject demographic characteristics are shown in Table 1.

**Table 1:** Demographic background information and control variable distribution characteristics of the research sample (*N* = 1,650).

Variable dimension	Statistical stratification	Sample size (*n*)	Proportion (%)
Country	China	907	55.0%
	Kazakhstan	743	45.0%
Gender	Female	906	54.9%
	Male	744	45.1%
Age distribution	20–29 years	249	15.1%
	30–39 years	666	40.4%
	40–49 years	492	29.8%
	50 years and above	243	14.7%
Highest education	Bachelor	170	10.3%
	Master	854	51.8%
	PhD	626	37.9%
Academic title	Assistant	315	19.1%
	Lecturer	659	39.9%
	Assoc. Prof	403	24.4%
	Professor	273	16.5%
Tenure	1–5 years	411	24.9%
	6–10 years	579	35.1%
	11–15 years	407	24.7%
	16 years and above	253	15.3%
Discipline	Science and Engineering (STEM)	577	35.0%
	Humanities	563	34.1%
	Medicine	169	10.2%
	Arts/Sports	153	9.3%
	Other	188	11.4%
Training time in the past year	0 h	325	19.7%
	1–10 h	672	40.7%
	11–20 h	402	24.4%
	Above 20 h	251	15.2%

### 3.2 Operationalization of measurement tools

The indicators for each dimension were contextually adapted based on mature scales. To coordinate with methodological error control, independent and mediating variables (digital self-efficacy, institutional support, technology anxiety) used a 5-point Likert scale (1 = “*strongly disagree*,” 5 = “*strongly agree*”); the dependent variable (technology integration behavior) used a 5-point frequency scale (1 = “*never*,” 5 = “*always*”). The complete original text of the measurement items, translated versions, and the operationalized data Codebook for each variable can be detailed in the supplementary materials of this study (Supplementary Files 1, 2).

#### 3.2.1 Digital self-efficacy (DSE)

Adapted based on the framework of Hatlevik (2017), reflecting individuals’ competency beliefs in mastering and applying complex digital tools (such as AI-assisted systems) in the absence of external immediate assistance. Specifically, the scale captures deeper cognitive layers beyond basic software navigation, evaluating teachers’ resilience and independent pedagogical troubleshooting capabilities. This unidimensional scale contains 5 items (e.g., “Even when facing complex new technologies, I believe I can master and apply them in the classroom”).

#### 3.2.2 Institutional support (IS)

A 5-item scale formed by borrowing the construct logic of “facilitating conditions” from Venkatesh et al. (2003). This construct aims to measure teachers’ perceived assessment of universities across comprehensive dimensions, encompassing not only physical hardware infrastructure but also intangible organizational climates, such as normalized technical mutual assistance, fault-tolerant culture, and administrative policy incentives (e.g., “The University regularly provides teachers with professional training opportunities regarding the application of educational technology”).

#### 3.2.3 Technology anxiety (TA)

Adopted the technostress scale from Ragu-Nathan et al. (2008). Distinct from basic operational worries, this scale focuses on the deeper psychological tension, cognitive overload, and emotional exhaustion experienced by teachers facing high-frequency technological iterations (5 items, e.g., “Because technology updates too fast, I feel stressed when using technology in teaching”).

#### 3.2.4 Technology integration behavior (TI)

Developed based on SAMR and DigCompEdu models, focusing on measuring the frequency of teaching behaviors evolving toward high-order integration. The scale focuses on the actual operation of using interactive media or intelligent tools to reconstruct classroom interaction patterns, specifically evaluating behavioral shifts from basic digital substitution to advanced pedagogical redefinition and student-centered knowledge co-creation (5 items, e.g., “Use advanced digital tools to reshape traditional classroom interaction links”).

#### 3.2.5 Control variables

To control for the confounding effects of individual backgrounds, the empirical model incorporated nationality, gender, age, highest education, academic title, tenure, and training duration in the past year as covariates into the analysis framework to ensure the accuracy of core path estimation.

### 3.3 Statistical analysis strategy

Data analysis utilized SPSS and Mplus software. Before entering core model construction, the distribution forms of observations were first evaluated through Skewness and Kurtosis tests. Given that large samples are often accompanied by a certain degree of normality deviation, parameter estimation uniformly used the robust Maximum Likelihood estimator based on Bootstrap resampling (MLR) to correct standard errors and ensure estimation robustness.

Data testing followed a nested logic: first, Confirmatory Factor Analysis (CFA) was used to evaluate the convergent and discriminant validity of core variables, and Harman’s single-factor test was introduced to screen for common method bias; subsequently, a Structural Equation Model (SEM) incorporating control variables was constructed to simultaneously estimate main effect associations, and combined with Bootstrap to test the indirect mediating role of technology anxiety.

To test the boundary conditions of the macro-environment, the model tested moderating effects by loading product interaction terms of psychological variables and the institutional environment. On the cross-national dimension, a Multi-Group Structural Equation Model (MG-SEM) was relied upon to construct equivalence constraint models to examine whether core paths possessed statistically significant structural heterogeneity between the teacher samples of the two countries.

## 4 Results analysis

### 4.1 Common method bias and measurement model testing

First, the distribution characteristics of the 20 observation items were examined. Absolute values of skewness for each item ranged from 0.052 to 0.399, and absolute values of kurtosis ranged from 0.643 to 0.902, showing no obvious univariate non-normality.

To screen for potential common method bias in self-reported data, this study used Harman’s single-factor test for evaluation. Unrotated principal component analysis extracted a total of 4 components with eigenvalues greater than 1. The first principal component explained 37.12% of the total variance, below the reference standard of 50%, indicating the study was less affected by common method bias. The KMO test result was 0.935, and Bartlett’s test of sphericity reached statistical significance (*χ*^2^ = 16,314.87, *p* < 0.001), indicating the data was suitable for factor analysis.

Subsequently, Confirmatory Factor Analysis was employed to test the four-factor measurement model consisting of digital self-efficacy, technology anxiety, institutional support, and technology integration behavior. This model contained 20 observation items. Results showed that the four-factor measurement model fit the data well: *χ*^2^ = 214.04, *df* = 164, *χ*^2^/*df* = 1.305, RMSEA = 0.013, CFI = 0.996, TLI = 0.996.

The reliability and convergent validity test results of the measurement model are shown in Table 2. Standardized factor loadings of each item ranged from 0.751 to 0.810, all reaching statistical significance. Cronbach’s α for each construct ranged from 0.865 to 0.888, and composite reliability also performed robustly, with AVE all above 0.50, indicating each construct had good internal consistency and convergent validity.

**Table 2:** Reliability and convergent validity test results of the measurement model.

Core construct	No. of items	Standardized factor loading range	Cronbach’s *α*	CR	AVE
Digital self-efficacy (DSE)	5	0.767—0.795	0.881	0.886	0.608
Technology anxiety (TA)	5	0.789—0.810	0.888	0.896	0.634
Institutional support (IS)	5	0.770—0.778	0.873	0.881	0.598
Technology integration behavior (TI)	5	0.751—0.767	0.865	0.873	0.578

CR, Composite Reliability; AVE, Average Variance Extracted.

Discriminant validity test results are shown in Tables 3, 4. The Fornell-Larcker criterion showed that the square roots of AVE for all constructs were greater than their correlation coefficients with other constructs. HTMT values ranged from 0.333 to 0.505, all below 0.85, indicating the four constructs had good discriminant validity.

**Table 3:** Fornell-Larcker discriminant validity test results.

Latent Variable	1	2	3	4
1. Digital self-efficacy (DSE)	**0.780**			
2. Technology anxiety (TA)	−0.276	**0.796**		
3. Institutional support (IS)	0.431	−0.176	**0.773**	
4. Technology integration behavior (TI)	0.476	−0.247	0.485	**0.760**

Bold values on the diagonal are the square roots of AVE for each construct; non-diagonal values are Pearson correlation coefficients between constructs.

**Table 4:** HTMT discriminant validity test results.

Latent variable	DSE	TA	IS	TI
DSE	—			
TA	0.465	—		
IS	0.431	0.333	—	
TI	0.493	0.425	0.505	—

All HTMT values are below 0.85.

### 4.2 Descriptive statistics and correlation analysis

Descriptive statistical results for the four core constructs are shown in Table 5. Digital self-efficacy had the highest sample mean (*M* = 3.495, *SD* = 0.971); the means for institutional support (M = 3.218, *SD* = 0.966) and technology integration behavior (*M* = 3.253, *SD* = 0.980) were relatively close; the mean for technology anxiety was close to the scale midpoint (*M* = 2.972, *SD* = 0.995). As shown in Figure 2, the distribution of digital self-efficacy is negatively skewed (with scores clustering at the higher end), whereas technology anxiety is positively skewed (with scores clustering at the lower end), and the distribution positions of institutional support and technology integration behavior are relatively close.

**Table 5:** Descriptive statistics and correlation matrix of core constructs and select covariates.

Dimension	*M*	SD	1	2	3	4	5 (Duration)	6 (Tenure)
1. DSE	3.495	0.971	1					
2. TA	2.972	0.995	−0.276***	1				
3. IS	3.218	0.966	0.431***	−0.176***	1			
4. TI	3.253	0.980	0.476***	−0.247***	0.485***	1		
5. Training duration	—	—	−0.001	0.018	0.006	0.009	—	
6. Tenure	—	—	−0.021	0.018	0.009	0.039		

DSE, Digital Self-Efficacy; TA, Technology Anxiety; IS, Institutional Support; TI, Technology Integration Behavior. Pearson correlation coefficients are reported among core constructs; Spearman rank correlation coefficients are reported between training duration, tenure, and other variables. ***p < 0.001.

**Figure 2:**
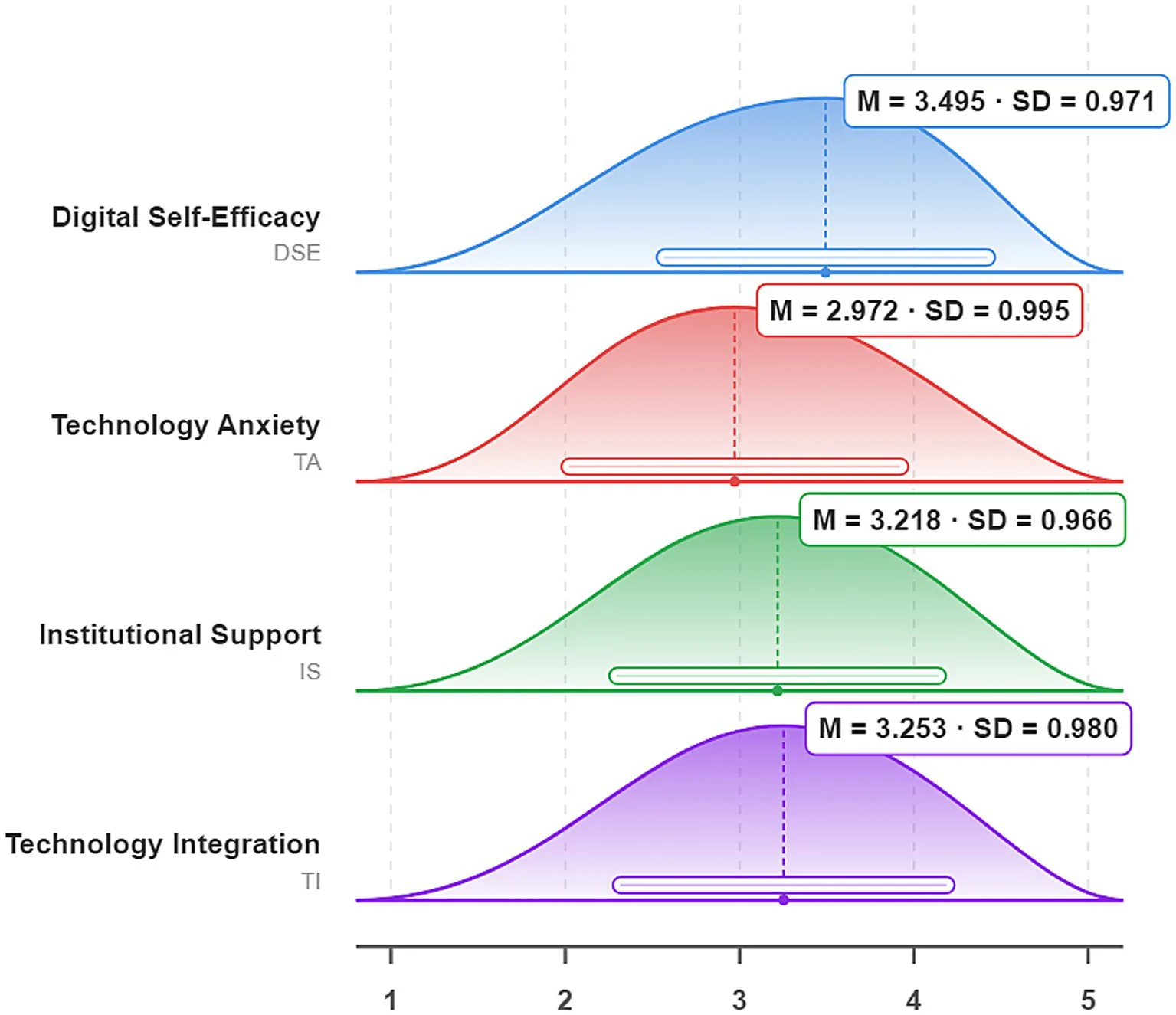
Kernel density ridge plot of composite scores of the four core constructs (*N* = 1,650). DSE, Digital Self-Efficacy; TA, Technology Anxiety; IS, Institutional Support; TI, Technology Integration Behavior. The composite score for each construct is calculated by averaging the scores of corresponding 5 items, with a theoretical range of 1—5. Vertical dashed lines represent sample means, and horizontal intervals represent one standard deviation above and below the mean.

Correlation analysis results are shown in Table 5. Technology integration behavior was significantly and positively correlated with digital self-efficacy (*r* = 0.476, *p* < 0.001) and institutional support (*r* = 0.485, *p* < 0.001), and significantly negatively correlated with technology anxiety (*r* = −0.247, *p* < 0.001). Digital self-efficacy was significantly negatively correlated with technology anxiety (*r* = −0.276, *p* < 0.001) and significantly positively correlated with institutional support (*r* = 0.431, *p* < 0.001). The directions of the above zero-order correlations were consistent with theoretical expectations but could not solely serve as evidence for mediating effects or causal relationships.

### 4.3 Path testing and interaction effects of the ecological mechanism model

Prior to full model testing, variance analysis and correlation analysis of demographic and teaching variables indicated that variables such as gender, age, and education level did not produce significant confounding interference on technology integration behavior. To maintain model parsimony, after finally controlling for the three covariates of nationality, tenure, and past training duration, this study adopted composite score path regression to test the associations among core constructs, with standard errors using HC3 robust estimation. The model’s explanatory power for technology integration behavior reached 30.5% (*R*^2^ = 0.305). The Variance Inflation Factors of core predictor variables ranged from 1.20 to 1.32, all far below 5.0, indicating the model had no obvious multicollinearity problems. The path structure diagram and test results of the model are shown in Figure 3 and Table 6.

**Figure 3:**
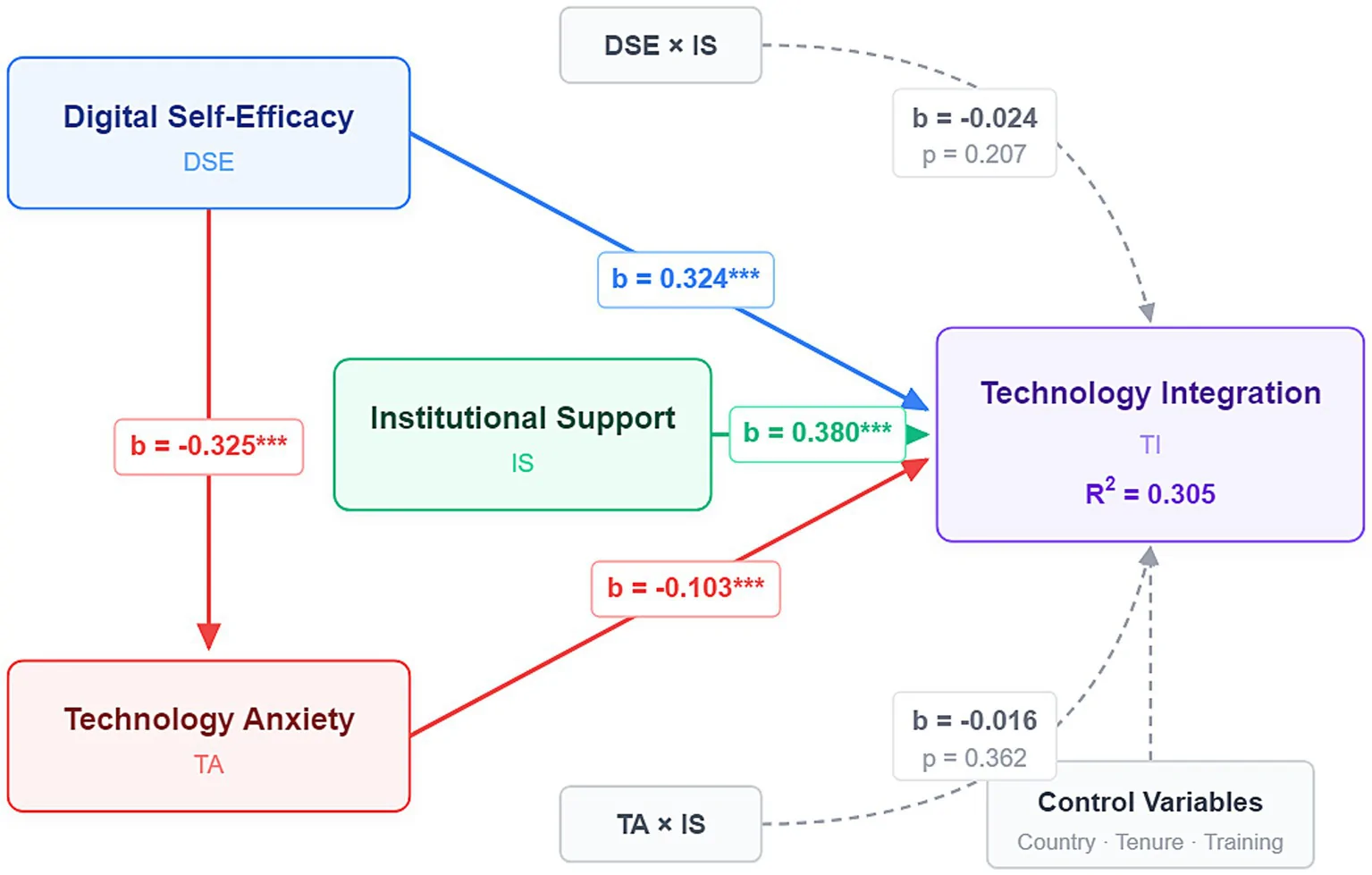
Ecological mechanism model of digital technology integration.

**Table 6:** Path test results of the structural equation model.

Path	*b*	*SE*	*z*	*p*	95% CI
DSE → TA	−0.325	0.029	−11.235	< 0.001	−0.381,−0.268
DSE → TI	0.324	0.030	10.718	< 0.001	0.265,0.383
TA → TI	−0.103	0.024	−4.345	< 0.001	−0.150,−0.057
IS → TI	0.380	0.030	12.577	< 0.001	0.321,0.439
DSE → TA → TI	0.034	0.009	3.778	< 0.001	0.018,0.052

DSE, Digital Self-Efficacy; TA, Technology Anxiety; IS, Institutional Support; TI, Technology Integration Behavior; *b*, Unstandardized path coefficient; CI, Confidence Interval. The model utilized MLR estimation and controlled for country, tenure, and past training duration. Indirect effects were tested using 5,000 Bootstrap resamples.

Digital self-efficacy and technology anxiety showed a significant negative association (*b* = −0.325, *SE* = 0.029, *z* = −11.235, *p* < 0.001), and a significant positive association with technology integration behavior (*b* = 0.324, *SE* = 0.030, *z* = 10.718, *p* < 0.001), supporting H1. Technology anxiety and technology integration behavior showed a significant negative association (*b* = −0.103, *SE* = 0.024, *z* = −4.345, *p* < 0.001). Bootstrap indirect effect testing showed that digital self-efficacy formed a significant positive indirect association with technology integration behavior via technology anxiety (*b* = 0.034, *SE* = 0.009, 95% CI 0.018, 0.052, *p* < 0.001). The confidence interval did not include 0, supporting H2. Furthermore, in exploring the peripheral system dimension, after stripping away psychological factors and control variables, institutional support still exhibited a strong and independent positive main effect association with technology integration behavior (*b* = 0.380, *SE* = 0.030, *z* = 12.577, *p* < 0.001), confirming the overall promotional expectation of a supportive environment on technological practice, supporting H3a.

When evaluating the boundary moderating characteristics potentially attached to the macro-environment (see Table 7), interaction testing was conducted after mean-centering the relevant participating variables. Model parameters showed that the interaction effect between digital self-efficacy and institutional support did not reach statistical significance (*b* = −0.024, *SE* = 0.019, *z* = −1.262, *p* = 0.207), nor did the interaction effect between technology anxiety and institutional support (*b* = −0.016, *SE* = 0.018, *z* = −0.911, *p* = 0.362). Therefore, institutional support did not significantly alter the association strength between digital self-efficacy or technology anxiety and technology integration behavior, thus neither H3b nor H3c were supported.

**Table 7:** Test results for institutional support interaction effects.

Interaction term	*b*	*SE*	*z*	*p*	95% CI	Hypothesis testing
DSE × IS	−0.024	0.019	−1.262	0.207	−0.062, 0.013	H3c not supported
TA × IS	−0.016	0.018	−0.911	0.362	−0.051, 0.019	H3b not supported

*b*, Unstandardized interaction effect coefficient; CI, Confidence Interval. Continuous variables participating in the interaction were all mean-centered. The model controlled for country, tenure, and past training duration, and standard errors used HC3 robust estimation.

### 4.4 Mean composite scores and model path differences in cross-national contexts

This study further compared the mean differences in composite scores of core constructs between the Chinese sample (*n* = 907) and the Kazakhstan sample (*n* = 743). Results are shown in Table 8. The two samples had no significant differences in digital self-efficacy (Δ*M* = 0.045, *p* = 0.356), technology anxiety (Δ*M* = −0.027, *p* = 0.580), and technology integration behavior (Δ*M* = 0.035, *p* = 0.472). The Kazakhstan sample scored higher than the Chinese sample on institutional support (Δ*M* = 0.094, *SE* = 0.048, *t* = −1.970, *p* = 0.049, Cohen’s *d* = 0.10). This indicates that teachers in both countries are relatively similar in digital self-efficacy, technology anxiety, and technology integration behavior, but exhibit certain differences in perceived institutional support, albeit with a notably small effect size.

**Table 8:** Mean difference test results for composite scores of core constructs in Chinese and Kazakh samples.

Core construct	China group *M*	Kazakhstan group *M*	Δ*M*	*t*	*p*
Digital self-efficacy (DSE)	3.475	3.520	0.045	−0.924	0.356
Technology anxiety (TA)	2.984	2.957	−0.027	0.553	0.580
Institutional support (IS)	3.175	3.269	0.094	−1.970	0.049
Technology integration behavior (TI)	3.237	3.272	0.035	−0.719	0.472

Δ*M* = *M*Kazakhstan − *M*China; a positive value indicates the Kazakhstan group mean is higher than the China group, and a negative value indicates it is lower; SE, Standard error of mean difference.

To further investigate whether model paths possessed cross-national differences, this study separately tested the four core paths in the Chinese and Kazakh samples and compared the corresponding regression coefficients. As shown in Figure 4, results are given in Table 9. Digital self-efficacy and technology anxiety showed significant negative associations in both the Chinese group (*b* = −0.282) and the Kazakhstan group (*b* = −0.282), with no significant inter-group difference (Δ*b* = 0.000, *p* = 0.985).

**Figure 4:**
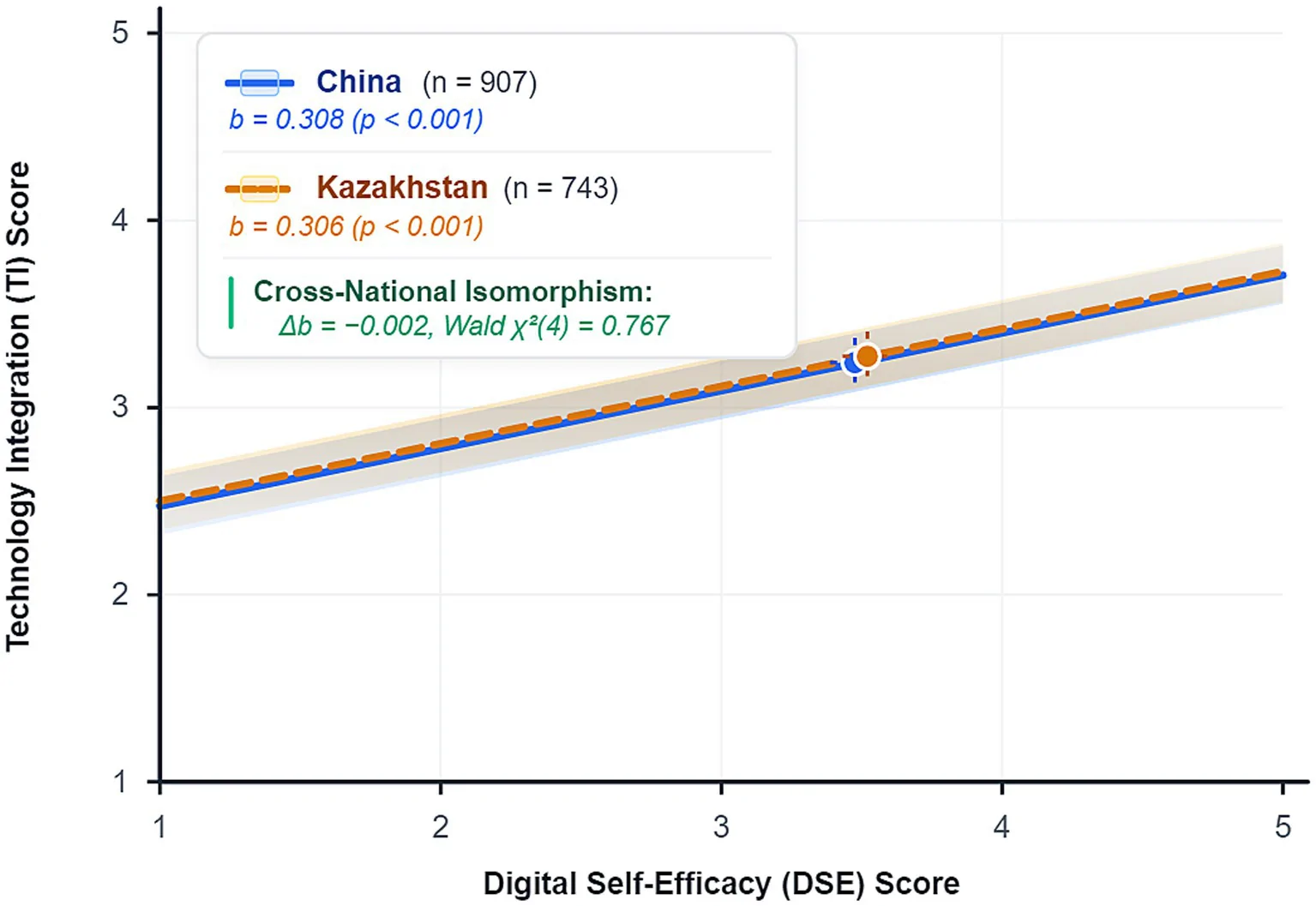
Cross-national structural isomorphism test of the covariant trajectories between core cognition and technology integration of teachers in China and Kazakhstan.

**Table 9:** Core path difference test results for Chinese and Kazakh samples.

Path	China group *b* (95% CI)	Kazakhstan group *b* (95% CI)	Δ*b*	*z*	*p*
DSE → TA	−0.282, −0.362,−0.202	−0.282, −0.369,−0.195	0.000	0.000	0.999
DSE → TI	0.308, 0.246,0.370	0.306, 0.232,0.380	−0.002	−0.045	0.964
TA → TI	−0.113, −0.166,−0.060	−0.093, −0.156,−0.031	0.020	0.460	0.645
IS → TI	0.357, 0.299,0.415	0.322, 0.251,0.393	−0.035	−0.744	0.457

DSE, Digital Self-Efficacy; TA, Technology Anxiety; IS, Institutional Support; TI, Technology Integration Behavior. *b* is unstandardized regression coefficient; Δ*b* = *b*Kazakhstan − *b*China; CI, Confidence Interval. Group analysis used the average score of the 5 items for each construct and controlled for tenure and past training duration; standard errors used HC3 robust estimation.

Digital self-efficacy and technology integration behavior showed significant positive associations in both the Chinese group (*b* = 0.308) and the Kazakhstan group (*b* = 0.306), with no significant inter-group difference (Δ*b* = −0.002, *p* = 0.941). Technology anxiety and technology integration behavior showed significant negative associations in both groups, similarly with no significant inter-group difference (Δ*b* = 0.020, *p* = 0.635). Institutional support and technology integration behavior showed significant positive associations in both groups, and their inter-group difference also did not reach statistical significance (Δ*b* = −0.035, *p* = 0.457).

The joint difference test for the four core paths did not reach statistical significance, Wald *χ*^2^(4) = 0.767, *p* = 0.943. Overall, the direction and strength of the four core paths were fundamentally consistent across the Chinese and Kazakh samples. Although there were differences in perceived levels of institutional support among teachers in the two countries, the associative structures among digital self-efficacy, technology anxiety, institutional support, and technology integration behavior did not exhibit significant cross-national heterogeneity.

To visually demonstrate this structural isomorphism more intuitively, Figure 4 presents the linear fitting trends between the core cognitive variable (digital self-efficacy) and explicit technological practice (technology integration behavior) for both samples. As shown in Figure 4, the regression slopes and confidence intervals of the Chinese group (solid blue line) and the Kazakhstan group (dashed orange line) highly overlap. This visual evidence further confirms that even subject to vastly different paces of macro-institutional evolution, the covariant pattern of teachers translating internal efficacy beliefs into technological behavior retains high cross-cultural invariance. Consequently, the exploratory hypothesis H4, which presumed inter-group heterogeneity, was not empirically supported.

## 5 Discussion

### 5.1 Theoretical interpretation of core findings: endogenous and exogenous mechanisms of higher education technology ecosystems

Previous studies often viewed the adoption of new technologies as a singular behavior, whereas this study confirmed through an ecosystem perspective that teachers’ level of digital practice is the result of consensual resonance between their internal psychological cognition and external institutional environment (Abbitt, 2011; Hatlevik, 2017). Facing the intrusion of generative artificial intelligence or complex adaptive learning systems, teachers with higher self-efficacy are more inclined to view them as cognitive resources extending teaching boundaries, and this intrinsic competency stably accompanies high-frequency technology integration practices.

Delving deeper along this psychological transmission link, technology anxiety is not a marginalized random deviation as in traditional models, but an inevitable negative emotional arousal for teachers coping with the human-machine collaboration transformation (Fettahoglu and Yikilmaz, 2025; Ragu-Nathan et al., 2008). Path estimations verified that high levels of self-efficacy can effectively stabilize evaluations of technological operations and significantly suppress technology anxiety. This emotional suppression effect indirectly paves a psychological buffer zone for technology implementation. This negative chain association indicates that the resistance to reconstructing teaching with technology is not just an operational barrier on a physical level, but externalizes as cognitive resource depletion resulting from individuals’ uncertainty (Mushtaque et al., 2022; Zhang, 2023). It is important to note that while our theoretical framework conceptually hypothesizes this sequentially directed mechanism—where self-efficacy actively mitigates anxiety to promote integration—the current cross-sectional data intrinsically captures this as an associational, rather than strictly causal, relationship. Nonetheless, empirically outlining this robust negative structural linkage (where high efficacy reliably co-occurs with low anxiety) remains theoretically paramount in validating our ecosystem view.

Transcending individual internal trait constraints, this study revealed the system-level function of institutional support. Unlike traditional contingency theories that set macro-environmental variables as conditional moderators (the interaction terms in this study were insignificant), institutional support presented a significant independent main effect in the current ecological model (Saleem et al., 2024). This implies that regardless of individual initial psychological baselines, the inclusive organizational network did not perform a selective buffering function merely for specific efficacy groups, but acts as an independent environmental variable that holistically elevates the digital operational level of the group. A thorough review of this non-significant moderating result reveals a profound theoretical insight: systemic institutional empathy and robust infrastructure are absolute prerequisites, not conditional supplements. It suggests that a supportive environment universally shifts the entire practical baseline upwards, rather than merely repairing the deficiencies of highly anxious individuals. This mechanism theoretically broadens the explanatory domain of the ecosystem approach; namely, by sharing the risks of trial and error, organizations construct an external support structure parallel to inner anxiety (Venkatesh et al., 2003).

In the multi-group validation, this study confirmed that this technology ecological structure possesses high cross-cultural invariance (no significant inter-group path differences). Samples from China and Kazakhstan exhibited high isomorphism on the chain where basic psychological feedback (efficacy and anxiety) drives technology integration. However, subject to objective differences in digital resource allocation and management systems, teachers’ perceived means of the institutional environment showed significant differences between the two countries. Such disparities reflect the distinct macro-stages of digitalization in the two countries. Specifically, the high-intensity advancement of higher education informatization in China often introduces performative evaluation pressures that may parallel resource provision, inadvertently diluting the perception of institutional inclusion. Conversely, the relatively early exploratory phase in Kazakhstan naturally offers a broader fault-tolerant space, effectively transmitting gentler environmental support signals to individuals. Nonetheless, given the notably small effect size calculated from the current data (Cohen’s *d* = 0.10), the practical significance of this systemic cross-national difference is somewhat limited. Therefore, these macro-policy interpretations should be treated as preliminary rather than definitive. Ultimately, these cross-national findings theoretically confirm that while the internal psychological mechanisms of navigating technology (from efficacy to anxiety) are inherently universal among humans, the external administrative friction that triggers these emotional responses is deeply sensitive to cultural and macro-policy regulations.

### 5.2 Practical implications: building sustainable digital partnerships in universities

The above empirical model not only completed the network validation of ecological theory but also provided administrators with operational coordinates for re-examining digitalization strategies. Reconstructing intervention strategies at the institutional level becomes the core pivot for breaking deadlocks of technological stagnation. Facing the evolution toward deep human-machine collaboration, university resource investment should move past the physical boundaries of procuring single software terminals and tilt toward constructing panoramic organizational support ecosystems. Such an architecture encompasses long-term peer mentoring networks, matched performance incentive restructuring, and crucially, the delineation of administrative fault-tolerant spaces. Only by accepting initial uncertainties at a cultural level can technological survival anxiety be diluted at the source (Bond et al., 2021).

Synergistically advanced interventions must also anchor onto the foundational reconstruction of the Continuous Professional Development (CPD) paradigm for teachers. Given the clearly presented covariant network in the data, continuous professional development programs should break away from purely technical operational training and systematically implant psychological efficacy building into the training process (Mekheimer, 2025; Siitonen et al., 2025). By implementing risk-free trial teaching programs, assisting teachers to accumulate positive feedback and translate it into internal efficacy during gradual operations, they can be prompted to cope with the digital environment with a more poised cognitive posture.

Scaling these practical insights beyond institutional boundaries, this study further abstracts a Global Digital Inclusion Policy Framework adaptable to the larger world educational population. Particularly for higher education systems in developing regions or emerging economies grappling with similar digital transition pains, macro-policymakers are advised to align with a universally scalable trilateral strategy. This framework mandates: (1) guaranteeing foundational infrastructural equity to bridge the digital divide between well-funded and grassroots institutions; (2) fostering culturally tailored socio-emotional safety nets (such as Kazakhstan’s transition-oriented fault-tolerance practices) rather than aggressive performance-driven mandates; and (3) initiating globally accredited, psychological resilience-focused professional development alliances. Implementing such a framework ensures that global macro-digitalization agendas progress synchronously with micro-level human wellbeing.

### 5.3 Research limitations and future directions

Constrained by the boundaries of the cross-sectional design, the generalization of these conclusions must consider the following limits. First, cross-sectional data only maps the covariant states among latent variables in a statistical sense; the current architecture cannot rule out potential reverse causality among variables (i.e., high-frequency practice might also conversely enhance digital self-efficacy). Establishing rigorous sequential evolutionary laws still relies on future longitudinal tracking designs.

Second, non-probability convenience sampling poses constraints on external validity. Subject to the potential structural interferences of disciplinary attributes (such as differences in laboratory dependence between arts and sciences) and university tiers, direct translation of the existing model to a full spectrum of educational institutions requires cautious academic reservation.

Finally, at the methodological and measurement levels, this study is subject to specific analytical compromises and potential reporting biases. On one hand, to ensure model convergence stability during multi-group invariance testing across two distinct large datasets, empirical testing employed composite score path regression (computing the mean of items). While practically effective, this approach falls short of a full Structural Equation Model (SEM) and cannot perfectly isolate measurement errors from structural paths. On the other hand, the exceptionally high descriptive response rate (94.3%)—largely facilitated through purposeful administrative distribution channels—raises concerns regarding Social Desirability Bias. Within such a professionally evaluated context, staff may harbor a latent tendency to positively skew or over-calibrate their technology integration practices to align with perceived institutional expectations. Future research should alleviate these constraints by deploying full-latent SEM architectures and cross-validating self-reports with objective Human-AI interaction logs extracted directly from Learning Management Systems (LMS).

## 6 Conclusion

Breaking away from a singular technology acceptance viewpoint, this study grounded itself in ecosystem theory and the socio-technical systems framework to systematically reveal the dual psychological and environmental driving mechanisms for advancing digital technology integration among university teachers in China and Kazakhstan. Empirical data confirmed that the effectiveness of teachers’ transformation toward high-order digital teaching is not only deeply rooted in internal digital self-efficacy but also negatively suppressed by technology anxiety. More crucially, regardless of micro-psychological traits, institutional support can act as an independent system-level resource to directly and significantly elevate the overall level of technology integration.

Furthermore, cross-national comparisons further confirmed that the psychological transmission link aimed at alleviating technology anxiety and promoting teaching innovation possesses high cross-cultural universality; however, the institutional fault-tolerant environment imprinted by macro-policy veins leads to uneven perceptions of the organizational inclusive atmosphere among teachers in different countries, albeit practically subtle. In summary, the digital transformation of higher education cannot solely rely on hardware accumulation or isolated skills training; it must move toward a sustainable ecological model driven by the dual wheels of “psychological adaptation-institutional inclusion.” The analytical framework and quantitative findings constructed in this study not only flesh out the theoretical map of educational technology ecosystems but also provide empirical coordinates that possess both universal laws and country-specific sensitivities for formulating intelligent transformation supporting policies in bilateral and multilateral higher education cooperation networks along the “Belt and Road” and beyond.

## Data Availability

The original contributions presented in the study are included in the article/Supplementary material, further inquiries can be directed to the corresponding author.
