# Supplementary material for: The ecological mechanism of digital technology integration: a comparative study of psychological and institutional support among university teachers in China and Kazakhstan
Source: Front Psychol. 2026 Sep 22;17:1959868. doi: 10.3389/fpsyg.2026.1959868 (PMC13638553; doi:10.3389/fpsyg.2026.1959868)
Supplement: Supplementary file 1 [file Data_Sheet_1.ZIP › Supplementary_File_2_Data_Codebook.docx]

Supplementary Material: Data Codebook

Dataset File: Dataset_N1650_China_Kazakhstan.csv

Sample Size: *N* = 1650 (China sample: *n* = 907; Kazakhstan sample: *n* = 743)

Data Preparation: The initial raw data was screened prior to analysis. As detailed in the manuscript, while approximately 7.8% of responses failed the reverse attention check, these cases were deliberately retained to maximally preserve the natural variance (Ecological Variance) of teachers facing high cognitive loads in real educational ecosystems, thus avoiding data homogenization caused by over-trimming. Only cases with patterned responses (e.g., straight-lining) and severe missing values were excluded to yield the final valid sample of 1,650 cases (effective recovery rate of 94.3%).

**Part 1: Demographic and Control Variables**

| Variable Name in CSV | Variable Label | Data Type | Coding Scheme / Values |
| --- | --- | --- | --- |
| Country | Nationality of the respondent | Categorical | 1 = China, 2 = Kazakhstan |
| Gender | Gender of the respondent | Categorical | 1 = Male, 2 = Female |
| Age | Age group | Ordinal | 1 = 20-29, 2 = 30-39, 3 = 40-49, 4 = 50+ |
| Education | Highest degree obtained | Ordinal | 1 = Bachelor, 2 = Master, 3 = PhD |
| Title | Academic Rank | Ordinal | 1 = Assistant, 2 = Lecturer, 3 = Assoc. Prof, 4 = Professor |
| Tenure | Teaching experience (Years) | Ordinal | 1 = 1-5 years, 2 = 6-10 years, 3 = 11-15 years, 4 = 16+ years |
| Discipline | Academic Discipline | Categorical | 1 = STEM, 2 = Humanities, 3 = Medicine, 4 = Arts/Sports, 5 = Other |
| Training_Hours | Tech-training hours (Past Year) | Ordinal | 1 = 0 hours, 2 = 1-10 hours, 3 = 11-20 hours, 4 = Above 20 hours |

**Part 2: Psychological and Environmental Constructs (Attitude Scale)**

Measurement Scale: 5-point Likert scale measuring the level of agreement.

[1 = *Strongly Disagree*, 2 = *Disagree*, 3 = *Neutral*, 4 = *Agree*, 5 = *Strongly Agree*]

| Construct | Item Code | Item Description (English Translation) |
| --- | --- | --- |
| Digital Self-Efficacy (DSE) | DSE1 | I am confident in my ability to use digital technology to solve various sudden difficulties encountered in teaching. |
|  | DSE2 | Even without the help of external technicians, I believe I can independently figure out and solve most software operation problems. |
|  | DSE3 | Faced with new educational needs, I have the ability to quickly learn and master a brand-new digital tool. |
|  | DSE4 | Even if the teaching environment changes drastically (e.g., a total shift to online), I am confident that I can ensure teaching quality using technology. |
|  | DSE5 | Even when facing complex new technologies (e.g., AI-assisted software), I believe I can master them and apply them to my classroom. |
| Institutional Support (IS) | IS1 | My university provides excellent technical infrastructure (e.g., internet, equipment) for teaching purposes. |
|  | IS2 | When I encounter technical failures in teaching, I can get timely help from specialized personnel. |
|  | IS3 | The university regularly provides professional training opportunities for teachers on the application of educational technology. |
|  | IS4 | The university has clear policies to encourage and reward teachers for using innovative technologies in teaching. |
|  | IS5 | The leadership is very supportive of my attempts to use new teaching technologies in the classroom. |
| Technology Anxiety (TA) | TA1 | I feel stressed when using technology in teaching because it updates so quickly. |
|  | TA2 | I worry about making mistakes and looking embarrassed when using technology in the classroom. |
|  | TA3 | I feel my workload has significantly increased in order to keep up with the development of educational technology. |
|  | TA4 | Using complex digital teaching tools makes me feel tense and uneasy. |
|  | TA5 | I am afraid that technology will gradually replace my leading role in the classroom. |
| Quality Control | ATT_CHK | Please select “Disagree” for this question to confirm you are answering carefully. (Reverse attention check item) |

**Part 3: Behavioral Construct (Frequency Scale)**

Note: To mitigate Common Method Bias (CMB) and establish discriminant validity against subjective psychological constructs, this dependent variable was operationalized using an objective behavioral frequency scale rather than an agreement scale.

Measurement Scale: 5-point Frequency scale evaluating actual teaching practices over the past semester.

[1 = *Never (0% of classes)*, 2 = *Rarely (~25%)*, 3 = *Sometimes (~50%)*, 4 = *Often (~75%)*, 5 = *Always (Almost every class)*]

| Construct | Item Code | Item Description (English Translation) |
| --- | --- | --- |
| Technology Integration (TI) | TI1 | Utilizing interactive digital media (rather than just static PPTs) to present multimedia teaching content in class. |
|  | TI2 | Assigning technology-based tasks that require students to use online collaboration tools (e.g., shared documents, virtual whiteboards) for group work. |
|  | TI3 | Tracking students’ learning data through the university’s Learning Management System (LMS) and providing personalized feedback accordingly. |
|  | TI4 | Integrating tasks into the course design that guide students to use specific digital web resources for inquiry-based learning, rather than just reading textbooks. |
|  | TI5 | Using advanced digital tools (e.g., instant polling apps, virtual simulations, or AI tools) to restructure traditional classroom interaction. |
