# Supplementary material for: The ecological mechanism of digital technology integration: a comparative study of psychological and institutional support among university teachers in China and Kazakhstan
Source: Front Psychol. 2026 Sep 22;17:1959868. doi: 10.3389/fpsyg.2026.1959868 (PMC13638553; doi:10.3389/fpsyg.2026.1959868)
Supplement: Supplementary file 1 [file Data_Sheet_1.ZIP › Supplementary_File_1_Questionnaire.docx]

### Survey on Digital Teaching Practices and Psychological Mechanisms among University Teachers

大学教师数字化教学现状与心理机制
Исследование цифрового преподавания и психологических механизмов преподавателей вузов

**Introduction & Instructions / 致谢与说明 / Введение и инструкции**

**English:**
Dear Teacher,
Hello! This questionnaire aims to investigate the psychological mechanisms and pedagogical behaviors of university teachers in China and Kazakhstan in the context of digital transformation. Your responses are crucial for improving the quality of higher education.
Confidentiality Guarantee: This survey strictly adheres to the principle of anonymity. All data will be used exclusively for academic statistical analysis and will never be used for any commercial purposes or personal performance evaluation. There are no right or wrong answers; please respond based on your actual situation.
Thank you for your support!

**中文：**
尊敬的老师：
您好！本问卷旨在研究中哈两国大学教师在数字化转型背景下的心理机制与教学行为。您的回答对提升高等教育质量至关重要。
郑重承诺：本调查严格遵循匿名保密原则，所有数据仅用于学术统计分析，绝不用于任何商业用途或个人考核。问卷无对错之分，请依据您的真实情况填写。
感谢您的支持！

**Русский:**
Уважаемый коллега! Данный опрос направлен на изучение психологических механизмов и педагогического поведения преподавателей вузов Китая и Казахстана в условиях цифровой трансформации. Ваши ответы крайне важны для повышения качества высшего образования.
Гарантия конфиденциальности: Опрос является строго анонимным. Все данные будут использованы исключительно для научного статистического анализа и никогда не будут переданы третьим лицам или использованы для коммерческих целей. Здесь нет правильных или неправильных ответов, пожалуйста, отвечайте искренне.
Спасибо за вашу поддержку!

#### Part I: Demographics (第一部分：背景信息 / Часть 1: Общая информация)

**Q1. Nationality / 您的国籍 / Ваше гражданство:**
[ ] China / 中国 / Китай
[ ] Kazakhstan / 哈萨克斯坦 / Казахстан

**Q2. Gender / 您的性别 / Ваш пол:**
[ ] Male / 男 / Мужской
[ ] Female / 女 / Женский

**Q3. Age / 您的年龄 / Ваш возраст:**
[ ] 20-29 years / 20-29岁 / 20–29 лет
[ ] 30-39 years / 30-39岁 / 30–39 лет
[ ] 40-49 years / 40-49岁 / 40–49 лет
[ ] 50 years and above / 50岁及以上 / 50 лет и старше

**Q4. Highest Degree / 您的最高学历 / Ваше высшее образование:**
[ ] Bachelor’s Degree / 学士 / Бакалавр
[ ] Master’s Degree / 硕士 / Магистр
[ ] Doctoral Degree (PhD) / 博士 / Доктор (PhD)

**Q5. Academic Rank / 您的职称 / Ваше ученое звание (должность):**
[ ] Teaching Assistant / 助教 / Ассистент
[ ] Lecturer / 讲师 / Преподаватель (Лектор)
[ ] Associate Professor / 副教授 / Ассоциированный профессор (Доцент)
[ ] Professor / 教授 / Профессор

**Q6. Teaching Experience / 您的教龄 / Ваш стаж преподавания:**
[ ] 1-5 years / 1-5年 / 1–5 лет
[ ] 6-10 years / 6-10年 / 6–10 лет
[ ] 11-15 years / 11-15年 / 11–15 лет
[ ] 16 years or more / 16年以上 / Более 16 лет

**Q7. Discipline Area / 您所属的学科领域 / Ваша дисциплинарная область:**
[ ] Humanities & Social Sciences / 人文社科 / Гуманитарные и социальные науки
[ ] STEM (Science, Engineering, Tech, Math) / 理工科 / Естественные и технические науки
[ ] Medicine / 医学 / Медицина
[ ] Arts & Sports / 艺术与体育 / Искусство и спорт
[ ] Others / 其他 / Другое

**Q8. Control Variable: Approximately how many hours of training related to educational technology have you attended in the past year?**
控制变量：在过去一年中，您大约参加了多少小时与教育技术相关的培训？
Контрольная переменная: Сколько часов обучения, связанного с образовательными технологиями, вы прошли за последний год?
[ ] 0 hours / 0 小时 / 0 часов
[ ] 1-10 hours / 1-10 小时 / 1–10 часов
[ ] 11-20 hours / 11-20 小时 / 11–20 часов
[ ] More than 20 hours / 20 小时以上 / Более 20 часов

#### Part II: Measurement Scales - Attitude and Psychological Assessment (第二部分：量表主体 - 态度与心理评估 / Часть 2: Основная часть - Оценка отношения и психологии)

**Scoring Instructions / 计分说明 / Инструкция по шкале:**
1 = Strongly Disagree / 完全不同意 / Полностью не согласен
2 = Disagree / 不同意 / Не согласен
3 = Neutral / 一般 / Нейтрально
4 = Agree / 同意 / Согласен
5 = Strongly Agree / 完全同意 / Полностью согласен

**A. Digital Self-Efficacy (数字自我效能感)**
**A1.** English: I am confident in using digital technologies to solve various unexpected difficulties encountered in teaching.
中文: 我有信心利用数字技术解决教学中遇到的各种突发困难。
Русский: Я уверен(а), что могу использовать цифровые технологии для решения различных непредвиденных проблем в обучении.
1 [ ] 2 [ ] 3 [ ] 4 [ ] 5 [ ]

**A2.** English: Even without the help of external technicians, I believe I can independently figure out and solve most software operation problems.
中文: 即使没有外部技术人员的帮助，我也相信自己能独立摸索并解决大部分软件操作问题。
Русский: Даже без помощи технических специалистов я верю, что смогу самостоятельно разобраться и решить большинство проблем с программным обеспечением.
1 [ ] 2 [ ] 3 [ ] 4 [ ] 5 [ ]

**A3.** English: Faced with new teaching needs, I am capable of quickly learning and mastering a brand new digital tool.
中文: 面对全新的教学需求，我有能力快速学会并掌握一种全新的数字工具。
Русский: Столкнувшись с новыми образовательными потребностями, я способен(на) быстро освоить новый цифровой инструмент.
1 [ ] 2 [ ] 3 [ ] 4 [ ] 5 [ ]

**A4.** English: Even if the teaching environment changes dramatically (such as a full shift to online teaching), I am confident that I can ensure teaching quality using technological means.
中文: 即使教学环境发生剧变（如全面转向线上），我也自信能用技术手段保障教学质量。
Русский: Даже при резком изменении условий (например, полном переходе в онлайн), я уверен(а), что смогу обеспечить качество обучения с помощью технологий.
1 [ ] 2 [ ] 3 [ ] 4 [ ] 5 [ ]

**A5.** English: Even when encountering complex new technologies (e.g., AI-assisted software), I believe I can master and apply them in my classroom.
中文: 即使面对复杂的新技术（如AI辅助软件），我也相信自己能够驾驭并应用到课堂中。
Русский: Даже сталкиваясь с новыми сложными технологиями (например, ПО с ИИ), я верю, что смогу их освоить и применить на занятиях.
1 [ ] 2 [ ] 3 [ ] 4 [ ] 5 [ ]

**B. Institutional Support / Facilitating Conditions (制度支持)**
**B1.** English: My university provides excellent technical infrastructure (e.g., internet, equipment) for teaching purposes.
中文: 我所在的大学提供了完善的技术基础设施（网络、设备）供教学使用。
Русский: Мой университет предоставляет отличную техническую инфраструктуру (интернет, оборудование) для преподавания.
1 [ ] 2 [ ] 3 [ ] 4 [ ] 5 [ ]

**B2.** English: When I encounter technical glitches in my teaching, I can get timely help from specialized personnel.
中文: 当我在教学中遇到技术故障时，能够及时获得专门人员的帮助。
Русский: Когда я сталкиваюсь с техническими сбоями, я могу быстро получить помощь от специалистов.
1 [ ] 2 [ ] 3 [ ] 4 [ ] 5 [ ]

**B3.** English: The university regularly provides professional training opportunities for teachers on the application of educational technology.
中文: 学校定期为教师提供关于教育技术应用的专业培训机会。
Русский: Университет регулярно предоставляет возможности профессионального обучения по применению образовательных технологий.
1 [ ] 2 [ ] 3 [ ] 4 [ ] 5 [ ]

**B4.** English: The university has clear policies to encourage and reward teachers for using innovative technologies in their teaching.
中文: 学校有明确的政策鼓励和奖励教师在教学中使用创新技术。
Русский: В университете существует четкая политика поощрения и награждения преподавателей за использование инновационных технологий.
1 [ ] 2 [ ] 3 [ ] 4 [ ] 5 [ ]

**B5.** English: The leadership is very supportive of my attempts to use new teaching technologies in the classroom.
中文: 领导层非常支持我在课堂上尝试新的教学技术。
Русский: Руководство активно поддерживает мои попытки использовать новые технологии на занятиях.
1 [ ] 2 [ ] 3 [ ] 4 [ ] 5 [ ]

**C. Technology Anxiety / Technostress (技术焦虑)**
**C1.** English: I feel stressed when using technology in teaching because technological updates happen too quickly.
中文: 因为技术更新换代太快，我在教学中使用技术时感到有压力。
Русский: Я чувствую давление при использовании технологий в обучении из-за их слишком быстрого обновления.
1 [ ] 2 [ ] 3 [ ] 4 [ ] 5 [ ]

**C2.** English: I worry that making a mistake while using technology in class will cause embarrassment.
中文: 我担心在课堂上使用技术时会出错，导致尴尬。
Русский: Я беспокоюсь, что при использовании технологий на уроке может произойти ошибка, что вызовет неловкость.
1 [ ] 2 [ ] 3 [ ] 4 [ ] 5 [ ]

**C3.** English: I feel my workload has significantly increased in order to keep up with the development of educational technologies.
中文: 为了跟上教育技术的发展，我感到工作负担显著增加。
Русский: Я чувствую, что моя рабочая нагрузка значительно возросла из-за необходимости следить за развитием образовательных технологий.
1 [ ] 2 [ ] 3 [ ] 4 [ ] 5 [ ]

**C4.** English: Using complex digital teaching tools makes me feel tense and uneasy.
中文: 使用复杂的数字教学工具让我感到紧张和不安。
Русский: Использование сложных цифровых инструментов обучения вызывает у меня напряжение и беспокойство.
1 [ ] 2 [ ] 3 [ ] 4 [ ] 5 [ ]

**C5.** English: I fear that technology will gradually replace my leading role in the classroom.
中文: 我害怕技术会逐渐取代我在课堂上的主导地位。
Русский: Я боюсь, что технологии постепенно вытеснят меня с лидирующей позиции в классе.
1 [ ] 2 [ ] 3 [ ] 4 [ ] 5 [ ]

**Check. Attention Check (质控题 / Контрольный вопрос)**
English: Please be sure to select “Disagree” for this question to confirm that you are reading carefully.
中文: 请务必在本题选择“不同意”，以确认您正在认真作答。
Русский: Пожалуйста, выберите “Не согласен” в этом пункте, чтобы подтвердить ваше внимание.
1 [ ] 2 [x] 3 [ ] 4 [ ] 5 [ ]

#### Part III: Behavioral Frequency (第三部分：实际应用频率 / Часть 3: Частота практического применения)

**Instructions / 操作说明 / Инструкция:**
English: Please recall your actual teaching practices over the past semester and evaluate how often you engaged in the following specific teaching behaviors.
中文: 请回忆您过去一个学期的实际教学情况，评估您从事以下具体教学行为的频率。
Русский: Пожалуйста, вспомните вашу реальную практику преподавания за последний семестр и оцените, как часто вы применяли следующие методы.

**Scale / 计分方式 / Шкала:**
1 = Never (从不 / Никогда: 0% of classes)
2 = Rarely (很少 / Редко: about 25% of classes)
3 = Sometimes (偶尔 / Иногда: about 50% of classes)
4 = Often (经常 / Часто: about 75% of classes)
5 = Always (总是 / Всегда: almost every class)

**D. Actual Technology Integration Behavior (实际技术融合行为)**
**D1.** English: I use interactive digital media (rather than just static PPTs) to present multimedia teaching content in the classroom.
中文: 在课堂上使用交互式数字媒体（而非仅展示静态PPT）来呈现多媒体教学内容。
Русский: Использование интерактивных цифровых медиа (а не только статических презентаций) для демонстрации учебного материала на занятиях.
1 [ ] 2 [ ] 3 [ ] 4 [ ] 5 [ ]

**D2.** English: I assign technology-based tasks that require students to use online collaboration tools (e.g., shared documents, virtual whiteboards) for group work.
中文: 布置以教育技术为基础的具体任务，要求学生利用在线协作工具（如共享文档、虚拟白板）进行小组合作。
Русский: Постановка задач на основе технологий, требующих от студентов использования инструментов для совместной работы в интернете (например, общих документов, виртуальных досок).
1 [ ] 2 [ ] 3 [ ] 4 [ ] 5 [ ]

**D3.** English: I track student learning data through the university’s online Learning Management System (e.g., LMS) and provide personalized feedback based on it.
中文: 通过学校的在线学习管理系统（如LMS系统等）跟踪学生的学习数据，并据此给予个性化反馈。
Русский: Отслеживание учебных данных студентов через систему управления обучением (LMS и др.) и предоставление на их основе индивидуальной обратной связи.
1 [ ] 2 [ ] 3 [ ] 4 [ ] 5 [ ]

**D4.** English: In course design, I guide students to use specific digital web resources for inquiry-based learning, rather than merely reading textbooks.
中文: 在课程设计中，引导学生使用特定的数字化网络资源进行探究式学习，而非仅仅阅读课本。
Русский: Интеграция в дизайн курса заданий, направляющих студентов на использование специфических цифровых ресурсов для исследовательского обучения, а не только чтение учебников.
1 [ ] 2 [ ] 3 [ ] 4 [ ] 5 [ ]

**D5.** English: I use advanced digital tools (e.g., real-time polling software, virtual simulations, or AI tools) to reshape traditional classroom interactions.
中文: 使用进阶数字工具（如即时投票软件、虚拟仿真或AI工具）来重塑传统的课堂互动环节。
Русский: Использование продвинутых цифровых инструментов (например, приложений для мгновенного голосования, виртуальных симуляций или ИИ-инструментов) для реструктуризации традиционного взаимодействия в классе.
1 [ ] 2 [ ] 3 [ ] 4 [ ] 5 [ ]
